# Device-compatible ultra-high-order quantum noise stream cipher based on delta-sigma modulator and optical chaos

**DOI:** 10.1038/s44172-024-00171-x

**Published:** 2024-02-07

**Authors:** Hanwen Luo, Ziheng Zhang, Longquan Dai, Linsheng Zhong, Qi Yang, Lei Deng, Deming Liu, Xiaoxiao Dai, Xiaojing Gao, Mengfan Cheng

**Affiliations:** 1 National Engineering Research Center for Next Generation Internet Access System, School of Optical and Electronic Information, Huazhong University of Science and Technology (HUST), Wuhan, 430074 China; 2 Jinyinhu Laboratory, Wuhan, 430048 China; 3 Shenzhen Huazhong University of Science and Technology Research Institute, Shenzhen, 518000 China; 4 The School of Computer Science, China University of Geosciences, Wuhan, 430074 China

**Keywords:** Fibre optics and optical communications, Optoelectronic devices and components

## Abstract

Data security is a key feature of future communications networks. Physical layer introduces rich physical mechanisms to increase the complexity of deciphering and provides extensive protection, but faces challenges in compatibility with commercial systems. Quantum noise stream cipher (QNSC) has been proposed as a promising solution to overcome this problem by fusing the stream cryptography regime with the quantum noise masking physical mechanism. However, it has limitations in terms of digital to analog conversion and clock data synchronization of ultra-high-order ciphertext as well as flexible control of masking noise. Here we report a 147.9-Gbps device-compatible quadrature amplitude modulation (QAM) QNSC secure scheme over 75-km fiber. Thanks to delta-sigma modulator, the transmission of 2^20^ × 2^20^-order QAM-QNSC signal are established through the low-order digital signal. We develop a theoretical model for flexibly regulating the transmission rate and security performance. Broadband optical chaos introduces true randomness and acts on the masking noise.

## Abstract (web summary)

Hanwen Luo and colleagues report a device-compatible ultra-high-order quantum noise stream cipher encrypted method. The approach flexibly regulates the data transmission rate and security performance of high-speed long-distance communications networks.

## Introduction

With the rapid development of big data and cloud computing, people are increasingly concerned about the security of private information. At present, sensitive private data is facing the threat of information monitoring, interception deciphering, and disguise attacks. The cryptographic algorithms based on computational time complexity have been applied to protect security. However, the rapid development of cryptanalytic deciphering technology and high-speed computing technology will reduce computational time and threaten cryptographic algorithms. Moreover, a thief stealing a digital key can go unnoticed^1^.

In order to deal with these threats, researchers turn to seek the possibility of establishing physical layer security. This security regime is built on the characteristics of physical devices. In the past two decades, various physical layer security schemes have been proposed and demonstrated, including quantum key distribution^2^, spread spectrum communication^3^, chaotic secure communication^4^, and optical steganography^5^, etc. Specifically, quantum key distribution joint one-time-pad can achieve absolute security. The spread spectrum communication has the advantage of anti-jamming. As for chaotic secure communication, it realizes security by scrambling the plaintext signal to a noise-like signal with broadband optical chaos. Optical steganography utilizes the stealth channel to transmit a secret message that is undetectable to the attacker. Those physical layer security schemes are still in the lab verification stage and not commercially available. The reason for this is that the compatibility of those regimes with the existing network faces several challenges. First, they need to deploy additional hardware devices (e.g., single-photon detector for quantum key distribution, chaos source for chaotic secure communication, amplifier spontaneous emission optical source for optical steganography). Second, security mechanisms based on physical characteristics cannot be directly integrated with the existing systems. Third, precise matching and regulation of analog keystreams to messages are difficult to achieve in the commercial system.

Quantum noise stream cipher (QNSC) is a method intending to provide secure communication by encrypting plaintext with stream cryptography and hiding the ciphertext with inevitable analog noise^6^. Specifically, the low-order modulated (LMF) plaintext is encrypted into extremely high-order modulated format (HMF) ciphertext under the control of the running key stream. After transmission, the adjusting levels of the ciphertext signal are masked by channel noise so that the eavesdropper cannot even get the correct ciphertext signal. This feature is distinctly different from other physical layer security solutions, as the QNSC scheme combines the failure of ciphertext interception with the complexity of cryptographic decipherment. One advantage of the digital stream cipher regime is compatible with the current system^7^. Meanwhile, the analog masking noise comes from the inevitable quantum noise in the transmission and does not require the deployment of additional hardware devices. In addition, the QNSC scheme requires only the matched digital keystream, so there is no need to consider the synchronization and matching of analog keystreams as in the above approaches. Hence, This excellent feature of the integration of digital and analog mechanisms has attracted much research and enabled the QNSC scheme to achieve ≥200 Gbps and ≥10,000 km application scenarios^8,9^.

Since the new approach to quantum cryptography was first proposed in ref. ^10^, QNSC is gradually gaining the attention of researchers. Based on the quantum detection and communication theory for classical channel, the users can employ encryption and decryption with the pre-shared secret key. The error performance of the attacker who does not have the key is worse than the legal users. This cryptography is called Y-00 protocol (so-called QNSC) since it is proposed by Yuen in 2000. In ref. ^11^, the experimental demonstration of the Y-00 protocol is carried out by an intensity-modulation scheme. After two decades of research, there have been proposed three main implementations of QNSC, including intensity-modulation QNSC, phase-modulation QNSC, and quadrature amplitude modulation (QAM) QNSC. As for the intensity-modulation QNSC, the implementation of encryption is to map the low-density multi-level plaintext signal to high-density multi-level ciphertext signal. The multi-level ciphertext signal is transmitted by intensity-modulation direct detection. In ref. ^12^, Yu et al. realized 100 Gbps intensity-modulation transmission over 100 km standard single-mode fiber (SSMF) with the help of single-sideband modulation and recursive least square algorithm used in Volterra equalizer. But the transmission distance of intensity modulation only reaches 100 km. Different from this, the implementation of phase-modulation QNSC is to rotate the phase of the plaintext signal. The phase-rotated ciphertext signal could be detected by digital-coherent detection, which decreases the power penalty of cipher system. This makes it possible to transmit over longer distance. So far, the longest transmission distance of phase-modulation QNSC scheme is 10,118 km^9^. But the transmission rate is limited to tens of Gbps. These two schemes cannot achieve both high speed and long distance at the same time. In ref. ^13^, Nakazawa et al. first proposed the QAM-QNSC and employed two-dimensional encryption (amplitude and phase) simultaneously for an enhancement of security. In ref. ^14^, 160-Gbps 16QAM-QNSC signal was transmitted over 320-km SSMF by an integrated two-segment silicon photonics I/Q modulator. In ref. ^8^, 201-Gbps signal QAM-QNSC signal was transmitted over 1200-km SSMF with probabilistic shaping. In summary, the applications of high-order modulated format, polarization division multiplexing, and digital coherent detection bring the QAM-QNSC scheme to the higher-speed and longer-distance system.

However, there are still several limitations to the further development of QNSC. First, the compatibility with the existing communication system has not been fully addressed. Although the QNSC scheme does not require additional hardware devices for noise masking, the transmission of the HMF ciphertext signals requires high-bit resolution and high-precision analog-to-digital converter and digital-to-analog converter (ADC/DAC), which will result in dramatic increases in deploy cost and implementation complexity. Some schemes have been proposed to handle this issue (e.g., coarse-to-fine modulation^15^). However, the usage of multiple DACs would also greatly increase the cost and system complexity. Second, the clock data recovery of HMF ciphertext signals is difficult. Among the current QNSC schemes, they all require an external clock channel to transmit the clock signals, which would increase the complexity of deployment. Third, the masking noise intensity cannot be flexibly regulated for the different scenarios. In the traditional QNSC schemes, the masking noise of the QNSC scheme comes from the quantum noise in the transmission link (e.g., the amplifier spontaneous emission noise of erbium-doped optical fiber amplifier (EDFA) and the shot noise of photodetector). The intensity of masking noise is adjusted by changing the magnification of EDFA and the receiving power of photodetector. But these devices are deployed at the transmission link and are inconvenient for adjustment. In summary, the study of QAM-QNSC scheme compatible with commercial devices is very necessary.

Here, we present a device-compatible QAM-QNSC scheme based on delta-sigma modulation (DSM) and optical chaos. The cooperation of the QNSC and DSM mechanisms makes it possible to transmit the HMF encrypted signal in LMF signal. The generation and reception of LMF signal does not need the high-performance DACs and ADCs. The implementation of QNSC encryption and delta-sigma conversion can be realized at the integrated circuit. Meanwhile, the modulation format of LMF signal is the same as the normal transmission system. Thus, the LMF signal could be transmitted through the current commercial transceivers without any modification. Moreover, the delivery and reception of LMF signal also makes it possible to achieve the clock data recovery of receiver in digital signal processing (DSP). Here, the quantization noise introduced by the DSM is used to mask the ciphertext signal. By scaling the oversampling zone, we can achieve flexible control of noise masking intensity for different security performance. Moreover, the scrambling of chaotic random number introduces the true randomness of optical chaos, which improves the randomness of masking noise. The true randomness introduced by optical chaos provides further randomization of the masking noise. The experimental system achieves 147.9-Gbps 64QAM-QNSC secure transmission supported by the single carrier 40 G-baud dual polarization (DP)16QAM over 75 km SSMF.

## Methods

### Flow chart of QAM-QNSC encryption with DSM

**Fig. 1:**
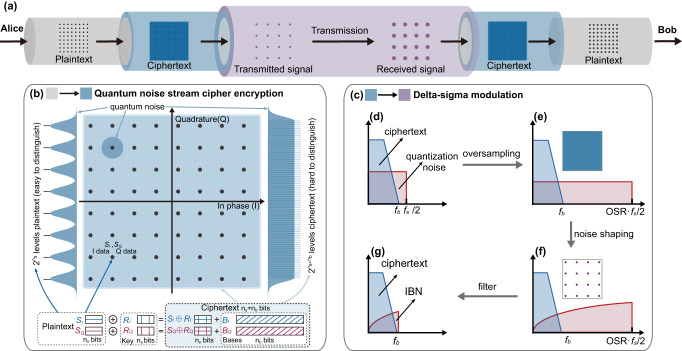
Scheme of quantum noise stream cipher (QNSC) with delta-sigma modulation (DSM). **a** Flow chart of the quadrature amplitude modulation (QAM) QNSC encryption and decryption with DSM. The QAM plaintext signal is encrypted into high-order QAM ciphertext signal by QNSC and converted into low-order QAM signal by DSM. After transmission, the received low-order QAM signal is restored to the high-order QAM ciphertext signal after a low-pass filter. Then, the plaintext signal is obtained by decrypting the ciphertext signal. **b** Two components of plaintext (*S*_*I*_, *S*_*Q*_) are encrypted by the random key (*R*_*I*_, *R*_*Q*_) and followed by bases (*B*_*I*_, *B*_*Q*_), respectively. Considering the quantum noise, sparse plaintext levels are easy to distinguish, but dense ciphertext levels are hard to distinguish. **c** High-order QAM ciphertext signal is converted into low-order QAM signal for transmission by DSM. **d** Ciphertext signal is sampled according to the Nyquist–Shannon sampling theorem. The half value of the sampling rate *f*_*s*_/2 is equal to or larger than the signal band *f*_*b*_ for the baseband signal. **e** Oversample the ciphertext signal can expand the sampling zone resulting in a lower floor of noise. **f** Noise shaping will push the quantization noise from low frequency to high frequency, leading to an uneven distribution of quantization noise. **g** Filtering out the out-of-band noise will get the ciphertext signal and in-band noise (IBN).

In Fig. 1a, the secure data transmission is established between Alice (transmitter) and Bob (receiver). At the transmitter, the binary data stream of Alice is first separated into two parallel data streams for the QAM modulation (I and Q component). After modulation, the plaintext is encrypted to ciphertext (HMF signal) through the running key of Alice. Then, the HMF signal are modulated to the LMF signal by DSM. After transmission, Bob obtains the transmitted LMF signal and recovers the HMF signal after a low-pass filter. Then, Bob obtains the plaintext signal by decrypting the original HMF signal with the pre-shared running key.

### Encryption and decryption of QNSC

In Fig. 1b, Alice gets the original QAM signal (*S*_*I*_, *S*_*Q*_) with the data length of *n*_*s*_ bits. The steps of the QNSC encryption are described as follows^13^: Step 1, the exclusive or (XOR) operation between the QAM signal (*S*_*I*_, *S*_*Q*_) and the random key (*R*_*I*_, *R*_*Q*_). Step 2, the base bits (*B*_*I*_, *B*_*Q*_) with the data length of *n*_*b*_ bits are added after the XOR signal. The random key and base bits come from the running key expanded from a seed key, which we assume is pre-share and secure. Hence, the HMF encrypted signal is with (*n*_*s*_ + *n*_*b*_) bits and could be written as:

1 $${(I,Q)}_{QNSC}=({S}_{I}\oplus {R}_{I}+{B}_{I},{S}_{Q}\oplus {R}_{Q}+{B}_{Q})$$

After transmission, the HMF encrypted signal is covered by quantum noise. Then, Bob performs the decryption operation, which is the reverse of encryption. After subtracting bases, Bob gets the encrypted signal (*S*_*I*_ ⊕ *R*_*I*_, *S*_*Q*_ ⊕ *R*_*Q*_). After demodulation, the plaintext signal is obtained by operating the XOR.

2 $${(I,Q)}_{Bob}=	 \ ({S}_{I}\oplus {R}_{I}\oplus {R}_{I},{S}_{Q}\oplus {R}_{Q}\oplus {R}_{Q})\\ =	\ ({S}_{I},{S}_{Q})$$

As for an eavesdropper (Eve), Eve could perform the same decryption operations. However, the running key of Eve is not the same as the running key of Alice. Hence, the encrypted signal is masked by the remaining bases and masking noise. In this situation, Eve even cannot obtain the correct ciphertext signal to decipher the message.

### Device-compatible transmission based on DSM

The HMF encrypted signal is transformed into the LMF signal by DSM, which has been widely investigated for fronthaul and access network applications^16–18^. The major feature is that the analog signal could be delivered through digital ports and be recovered using only a filter. The principle of DSM in the frequency domain is illustrated in Fig. 1c. As for the Nyquist ADCs shown in Fig. 1d, the half value of the sampling rate *f*_*s*_/2 is equal to or larger than the signal band *f*_*b*_ for the baseband signal. During the conversion of the analog waveform to a digital signal, the quantizer will introduce quantization noise, which is uniformly distributed over the entire Nyquist sampling area^19^. As for the DSM, the first step is to oversample the signal to expand the sampling zone as shown in Fig. 1e. The quantization noise spreads over a wide frequency range and the floor of it is reduced. The second step is noise shaping. It would push the quantization noise from low frequency to high frequency. More quantization noise is squeezed out of the signal band, leading to the uneven distribution of quantization noise as shown in Fig. 1f. The in-band noise (IBN) is furtherly reduced. Thus, the HMF signal can be converted to the LMF signal with only one or two quantization bits. At the receiver side, the original HMF signal can be easily retrieved by filtering out the out-of-band noise as shown in Fig. 1g^20^. The receiving analog signal consists of the whole original signal and the IBN.

**Fig. 2:**
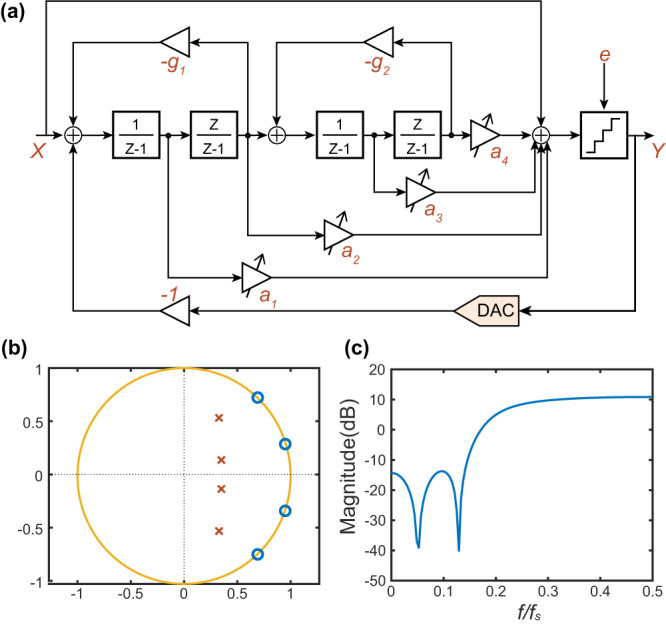
Design of delta-sigma modulator (DSM). **a** The structure of 4-order DSM based on cascade-of-resonators feedforward structure; **b** the zero-poles plot and **c** noise transfer function curve. The parameters *a*_*1-4*_ of DSM structure are controlled by optical chaos.

Here, we consider a fourth-order DSM based on a cascade-of-resonators feedforward structure as shown in Fig. 2a. The loop-filter consists of two nondelaying integrators and two delaying integrators. Each integrator output is connected to the input of the quantizer with different weight factors *a*_*1-4*_. The resonators are created by the internal feedback from the output of one delaying integrator to the input of the front nondelaying integrators with different feedback weight factors *g*_*1-2*_. The output *Y* of the quantizer is fed back to the input X of the whole structure. *e* is the quantization noise. Analysis of Fig. 2a in *z*-domain yields is written as:

3 $$Y(z)=STF\cdot X(z)+NTF\cdot e(z)$$

According to Zhong et al.^21^, the noise transfer function (NTF) and signal transfer function (STF) of the fourth-order DSM structure could be written as follows:

4 $$\frac{1}{NTF}=1+\frac{{a}_{1}(z-1)+{a}_{2}z}{{(z-1)}^{2}+{g}_{1}z}+\frac{[{a}_{3}(z-1)+{a}_{4}z]z}{[{(z-1)}^{2}+{g}_{1}z][{(z-1)}^{2}+{g}_{2}z]}$$

5 $$STF=1$$

6 $$z={e}^{\frac{j2\pi f}{{f}_{s}}},f\in [0,{f}_{s}/2]$$

Where *f* is a frequency variable, and *f*_*s*_ is the sampling rate of DSM.

A typical set of DSM parameters generated by the Delta Sigma Toolbox of MATLAB^22^ are given in Table 1. The NTF design code can be found in the supplement file. The zero-poles plot is given in Fig. 2b. As for the zero-poles plot, optimally spreading the zeros can reduce the noise power of IBN. Figure 2c shows the magnitude of the fourth-order NTF. The low frequency of the NTF curve is lower than the high frequency.

**Table 1:** Typical parameters of fourth-order DSM.

*a*_*1*_	*a*_*2*_	*a*_*3*_	*a*_*4*_	*g*_*1*_	*g*_*2*_
0.9450	0.9798	0.3153	−0.3442	0.1009	0.6181

### Theoretical model of masking noise

The IBN plays the role of masking noise in this scheme. Here, we derive the relationship between the masking noise intensity and the oversampling zone of DSM. Further, we obtain a theoretical model for flexibly regulating the security performance of the system. Moreover, we proposed a randomness enhancement method by scrambling the parameters of NTF with chaotic random number sequence.

The oversampling rate (OSR) is determined by the signal bandwidth *f*_*b*_ and the sample rate *f*_*s*_. The theoretical model of IBN can be written as^23^:

7 $$OSR=\frac{{f}_{s}}{2{f}_{b}}$$

8 $$IBN=\frac{{\delta }^{2}}{12\pi }{\int }_{0}^{\frac{\pi }{OSR}}{|NTF({e}^{j\omega })|}^{2}d\omega$$

Here, *δ* is the quantization interval. *π*/*OSR* is the signal band. According to Eqs. (7) and (8), a high OSR will lead to a low IBN, resulting in a better transmission quality of analog signal. Thus, for the application of transmitting the high-precision lossless analog signal, a larger oversampling is applied to distribute the quantization noise over a wide frequency range and get a lower floor of the IBN. Different from this, in this scheme, we intentionally lower the OSR to increase the IBN intensity for a better masking performance. The number of masked signals (NMS) is used to represent the security performance of QAM-QNSC system. The estimation method for NMS is defined as^13^:

9 $${{{{{\rm{NMS}}}}}}={\varGamma }_{I}{\varGamma }_{Q}=\left(\frac{2{\bar{\sigma }}_{I}}{\varDelta }\right)\left(\frac{2{\bar{\sigma }}_{Q}}{\varDelta }\right)$$

10 $${\bar{\sigma }}_{I,Q}=\sqrt{\frac{1}{2n}\mathop{\sum }\limits_{1}^{n}({\sigma }_{I,n}^{2}+{\sigma }_{Q,n}^{2})}$$

Here, Γ_*I*_ and Γ_*Q*_ are the NMS of I/Q component. Δ is the interval of two adjacent levels. σ_*I,Q*_ is the standard deviation of noise in every constellation point. *n* is the number of ciphertext states.

According to Eqs. (8) and (9), it presents that the NMS increases as the OSR increases. When the NMS is larger, the number of signals that the eavesdropper needs to distinguish is more, and the possibility that the eavesdropper obtains the correct ciphertext is lower. Hence, different security performances can be realized by adjusting the OSR for various scenarios.

### Security improvement with chaotic random scrambling

**Fig. 3:**
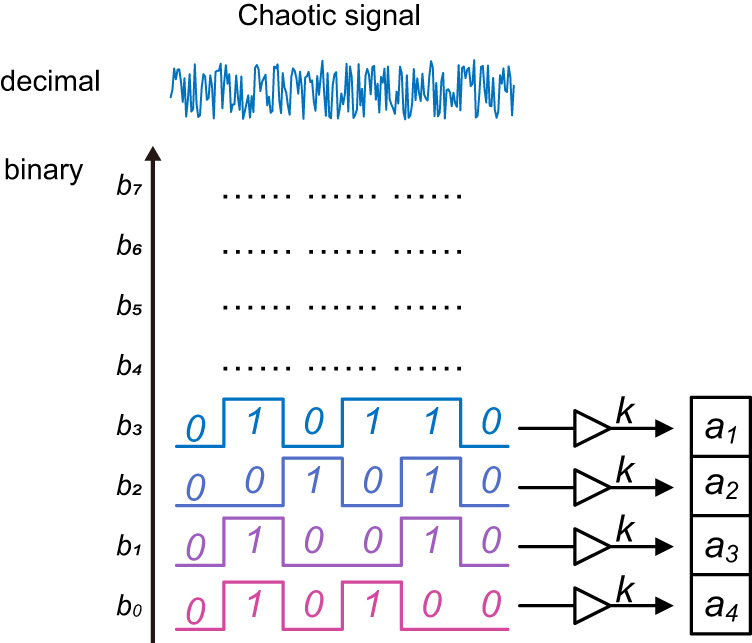
Chaotic random number sequences scramble delta-sigma modulator parameters. Every bit of the optical chaotic signal can be considered as an independent sequence of random numbers and used to continuously scrambling the weight factors *a*_*1-4*_.

However, the IBN is determined by the input signal and NTF, so the IBN can be considered as a pseudo-random sequence and definite for the same input sequence and a fixed DSM^24^. This is a potential concern for the system security. Here, we continuously change the parameters of the DSM through a chaotic random number sequence to improve the randomness of IBN. The random number sequence is generated from an optical chaotic signal by extracting different bits of an 8-bit digital quantizer. Every bit of the optical chaotic signal can be considered as an independent sequence of random numbers. We use the random number sequence continuously scrambling the parameters of NTF, as shown in Fig. 3. The scrambling will lead to a changing NTF so that the IBN will be more random. According to Eq. (4), the NTF can be rewritten as follows:

11 $$\frac{1}{NTF}=1+\frac{{a{\prime} }_{1}(z-1)+{a{\prime} }_{2}z}{{(z-1)}^{2}+{g}_{1}z}+\frac{[{a{\prime} }_{3}(z-1)+{a{\prime} }_{4}z]z}{[{(z-1)}^{2}+{g}_{1}z][{(z-1)}^{2}+{g}_{2}z]}$$

12 $${a{\prime} }_{i}(t)={a}_{i}+k\cdot {b}_{4-i}(t),i\in \{1,2,3,4\}$$

Here, *a*_*i*_ is the original parameters. *b*_*i*_ is the random number sequence generated from different bit position. *k* is the scrambling weight.

According to the analysis above, the randomness of masking noise of the same input signals can be improved by the random number sequence scrambling. We assume that the scrambling is applied to a segment length of the signal. The scrambling rate is equal to the signal rate divided by the segment length. However, during the segment length, the parameters of DSM remain the same. It is possible to crack the parameters of DSM if the segment length is long enough. Hence, the segment length should be as short as possible, and the scrambling frequency should be as fast as possible. When the scrambling rate is consistent with the signal rate, the continuously changing input signal and DSM parameters would make deciphering difficult to complete. The mixing property of chaos can transduce the nondeterministic microscopic noise to nondeterministic macroscopic states for the generation of truly random sequences^25^. Optical chaos allows for high bandwidth and thus meets the high-rate requirements of random number scrambling. The true randomness is introduced to the masking noise by the random number sequence extracted from optical chaos acting on the DSM parameters. Thus, the security enhancement is achieved by indirectly introducing the true randomness of optical chaos.

### Experimental setup

**Fig. 4:**
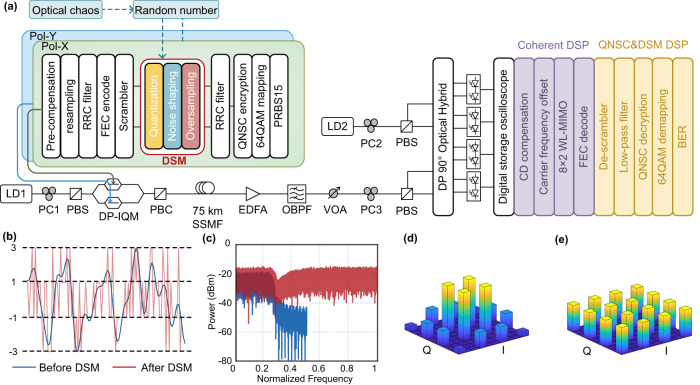
Experimental setup. **a** PRBS15: pseudo-random binary sequence, QNSC: quantum noise stream cipher, RRC: root-raised-cosine filter, DSM: delta-sigma modulator, FEC: forward error correction, Pol-X/Y: polarization X/Y, LD: laser diode, PC: polarization controller, PBS: polarization beam splitter, DP-IQM: dual polarization I/Q modulator, PBC: polarization beam combiner, SSMF: standard single-mode fiber, EDFA: erbium-doped optical fiber amplifier, OBPF: optical band-pass filter, VOA: variable optical attenuator, CD compensation: chromatic dispersion compensation, WL-MIMO: widely-linear multiple-input and multiple-output, BER: bit error ratio. **b** Time domain waveform and **c** spectra of the I component signal before (blue line) and after (red line) DSM. Constellation of the 16-quadrature amplitude modulation (QAM) signal **d** before and **e** after the scrambler.

The experimental setup of QAM-QNSC encryption and decryption with DSM and optical chaos is shown in Fig. 4a. At the transmitter (Alice), the laser diode (LD1, Alnair Labs, TLG-200) emits a 15 dBm optical carrier with a wavelength of 1550.001 nm and linewidth of ~100 kHz. A polarization controller (PC1) is aligned at 45° angle to the polarization beam splitter (PBS). Then, the light is modulated in the dual polarization I/Q modulator (DP-IQM, Fujitsu, FTM7977HQ/611). The 40Gbaud DP-16QAM signal is generated by a 64-GSa/s arbitrarily waveform generator (Keysight M8195A) with 8-bit vertical resolution. The polarization-X and polarization-Y data of the DP-16QAM signal are generated by the DSP module in MATLAB R2020a.

Here, we take the polarization-X data as an example. First, the plaintext sequence is generated from the sequence of pseudo-random binary sequence (PRBS15) with a length of 2^15^-1. After modulation, the plaintext sequence is modulated to the 64QAM signal. Then, the 64QAM plaintext signal is encrypted to 2^20^ × 2^20^ QAM ciphertext signal according to the QNSC protocol. After the root-raised-cosine filter, the I and Q components of the ciphertext signal pass through the DSM respectively. A roll-off factor of 0.1 was used. The parameters of DSM are generated by the Delta Sigma Toolbox^22^ and scrambled by the chaotic random number sequence, which is generated by our previous work^26^. This chaos source consists of an optoelectronic oscillator based on commercial devices, which can achieve a bandwidth of 29.1 GHz (10 dB bandwidth). Figures 4b, c show the time domain waveform and the spectra of the I component signal before and after the DSM. The analog-like ciphertext signal is plotted in blue, and the PAM4 signal is plotted in red. In Fig. 4b, the ciphertext signal is quantized to the PAM4 signal, where the difference is the quantization noise. For a clearer visual presentation of the system’s behavior, we observe the input and output signal in the frequency domain, as shown in Fig. 4c. We can see that the signal spectrum remains unchanged after DSM, and the quantization noise has an uneven distribution. More quantization noise is pushed out of the signal band. After DSM, we can find that there are more ±1 symbols than ±3 symbols in the red curve of Fig. 4b. This reason is that the ciphertext signal after the root-raised-cosine filter has an unequal distribution. So, the 16QAM signal has the same unequal distribution on its constellation, as shown in Fig. 4d. The traditional coherent algorithms cannot deal with the unequally distributed constellation. To equalize the constellation, we insert a scrambler in the transmitter. The constellation of the 16QAM signal after the scrambler is shown in Fig. 4e. Then, forward error correction (FEC) of the Reed-Solomon code is used with a code rate of 239/255. For a high baud rate transmission, the impairment calibration of the band-limited device is indispensable^27^. We pre-compensate the amplitude and frequency response and the IQ/XY skew at the transmitter using the method of ref. ^28^.

Then, the DP-16QAM signal is transmitted over 75 km SSMF, whose attenuation is 0.2 dB/km. To compensate for the power loss, the DP-16QAM signal is amplified by the EDFA (Amonics, AEDFA-CDWDM-23-B-FA) and passes through an optical band-pass filter (OBPF, Yenista XTM-50) reducing the output noise of EDFA.

At the receiver (Bob), a variable optical attenuator (VOA) is used to adjust the received optical power (ROP) not to exceed −10 dBm. The local light is emitted from another laser diode (LD2) with an optical power of 10 dBm and a wavelength of 1550.001 nm. The PC2 and PC3 are also aligned at 45° angle to the PBSs. The coherent receiver consists of a DP 90° optical hybrid and four balanced photodetectors. The four-channel received electrical signals are sampled and digitalized by a 50-GSa/s digital storage oscilloscope (Tektronix, DPO73304D). Then, the received digital signal is processed offline in MATLAB R2020a.

It is worth mentioning that the clock data can be easily recovered by the clock data recovery algorithm. Since the transceiver receives low-order digital signals (four-level), the clock signal can be extracted through the clock data recovery algorithm^29^. It would eliminate external clock lines or tone clock signals and could greatly simplify the system. The DSP consists of two parts: the coherent DSP and the QNCS&DSM DSP. The former is used to equalize and compensate for the impairments in the system for the DP-16QAM signal. The latter is to recover the ciphertext and decrypt it for plaintext. In the former, the chromatic dispersion (CD) compensation and carrier frequency offset estimation are implemented first^30^. After that, an 8 × 2 widely-linear multiple-input and multiple-output (WL-MIMO) algorithm is introduced to implement the channel equalization, polarization demultiplexing, and the transmitter impairment post-compensation. The tap coefficient is 63, the step size is 1.2 × 10^−5^, the step size of phase is 64 and the overhead 5000 16QAM symbols are for pre-convergence of the MIMO equalizer^31^. Then, the error symbol is corrected by FEC. In the latter, the QNSC encrypted signal is recovered through a de-scrambler and low-pass filter, followed by the QNSC decryption, 16QAM de-mapping, and bit error rate (BER) calculation in turn.

## Results and discussion

### The BER of 64QAM plaintext and NMS with different OSRs when the transmission of DP-16QAM is error-free

**Fig. 5:**
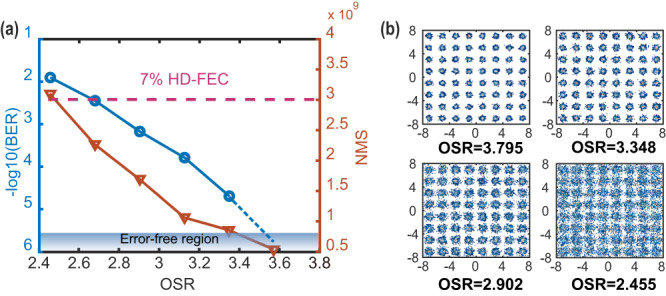
The bit error ratio (BER) of 64-quadrature amplitude modulation (QAM) plaintext and number of masked signals (NMS) with different oversampling ratios (OSR). **a** The BER of plaintext and the NMS versus OSR after quantum noise stream cipher decryption. The dotted line represents the hard-decision forward error correction (HD-FEC, −log10(BER) = 2.42) with an overhead of 7%. **b** Constellations of 64QAM signal with different OSRs.

The plaintext BER is related to the signal-to-noise ratio (SNR), which is only determined by IBN. According to Eq. (8), IBN is inversely correlated with the OSR. Hence, the SNR is positively correlated with OSR. Figure 5a shows the BER of 64QAM plaintext signal and NMS under different OSRs (raw data see Supplementary Data 1). The decryption BER and NMS decrease when the OSR increases since a higher OSR will reduce the noise floor. The drop of NMS would bring a weaker masking performance. Meanwhile, the bitrate of plaintext would decrease as the OSR increases since the signal bandwidth becomes narrow. In fact, a higher OSR (higher SNR) can support a higher modulation format plaintext signal transmission (higher bitrate). The detail discussion will be discussed later. Figure 5b shows the constellation diagram of the 64QAM signal at different OSRs. We can find that the constellation becomes indistinct when the OSR decreases. The minimum required OSR is 2.679 when the BER arrives at the hardware forward FEC threshold. If we consider 7% HD-FEC (hard decision, BER = 3.8 × 10^−3^) overheads, the net plaintext data rate is (40 G-Baud × 6 bit/symbol × 2 pol.)/(2.679 OSR)/(1 + 6.2% FEC for 16QAM + 7% FEC for 64QAM + 8% pilot) = 147.9 Gbps. Plaintext data rate can be further increased by setting the OSR as low as possible, decreasing the overhead, and achieving a higher DP-16QAM transmission speed.

### The influence of error symbols that occur in the transmission of DP-16QAM on 64QAM plaintext

**Fig. 6:**
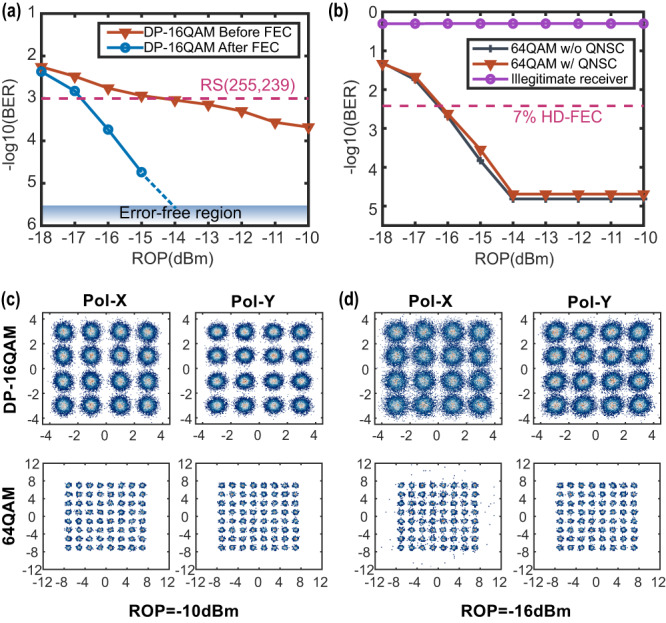
The transmission performance of dual-polarization quadrature amplitude modulation (DP-QAM) 16QAM signal and its influence on 64QAM signal. **a** The bit error ratios (BER) of 16QAM signal before and after forward error correction (FEC, RS(255,239), −log10(BER) = 3) under different received optical powers (ROP). **b** BERs of 64QAM signal with and without quantum noise stream cipher (QNSC) encryption versus different ROPs. The dotted line represents the hard-decision FEC (HD-FEC, −log10(BER) = 2.42) with an overhead of 7%. (**c**) and (**d**) show the polarization X (Pol-X) and polarization Y (Pol-Y) constellation diagram of DP-16QAM signal and 64QAM signal at different ROPs.

The transmission performance of the DP-16QAM signal fluctuate because of unavoidable optical power loss. Thus, we investigate the BER of the DP-16QAM signal under the back-to-back experiment system, as shown in Fig. 6 (raw data see Supplementary Data 2). Without loss of generality, the OSR is set to 3.348 for the following results. In Fig. 6a, we study the BER of the DP-16QAM signal under different ROPs and show the effect of FEC with RS (255,239). The BER of the DP-16QAM signal before FEC drops with the increase of ROP. When the raw BER reaches up to 1 × 10^−3^, the error symbols can be fully corrected by employing the FEC. The minimum ROP is −14 dBm for an improved error-free transmission. Furtherly, we study the sensitivity of the 64QAM signal to the error symbol of the DP-16QAM signal, as shown in Fig. 6b. When ROP is above −14 dBm, no error symbol appears, so the BER of the 64QAM signal maintains a constant equal to 1.53 × 10^−5^. When the ROP decreases, degraded transmission quality of the DP-16QAM signal causes symbol errors, resulting in the rise of 64QAM signal BER. The constellation diagrams of the DP-16QAM signal and 64QAM signal are given in Fig. 6c, d. When the ROP is −10 dBm, the constellation of the DP-16QAM signal is clear. When the ROP is −16 dBm, the constellation of the DP-16QAM signal becomes hard to distinguish. The error symbols of the DP-16QAM signal will be reflected on the 64QAM signal and become external noise. This noise will lead to some constellation points of the 64QAM signal running out of the standard zone. The variations between channels Pol-X and Pol-Y come from the discrepancy between the output channel of AWG and the noise of the receiver. Therefore, the error-free transmission of DSM-supported DP-16QAM is critical in this scheme.

### Encryption penalty and the BER comparison between legal users and illegal users

We compare the BER of 64QAM signal with (red line) and without (black line) QAM-QNSC encryption in Fig. 6b. The disparity between red line and black line is very small, meaning the encryption penalty is slight. However, the encryption penalty of the traditional QNSC system is unavoidable for about 1 dB because the effective numbers of bits of the DAC/ADC are less than the resolution and the higher order signal is more sensitive to the nonlinearity of drivers and TIAs^32^. Moreover, we discuss the situation of tapping attack from an illegitimate receiver (Eve). We assume that Eve has access to the transmission DP-16QAM signal with coherent DSP. Then, in the QNSC-DSM DSP section, Eve can filter out the out-band noise and obtain the ciphertext signal covered by masking noise. Finally, Eve attempts to decipher the QNSC ciphertext signal without the correct key stream. The BER (purple line) of the illegitimate receiver stays around 0.5, as shown in Fig. 6b. The high BER shows that the security of system is guaranteed.

### Dynamic control of security performance  and plaintext data rate

**Fig. 7:**
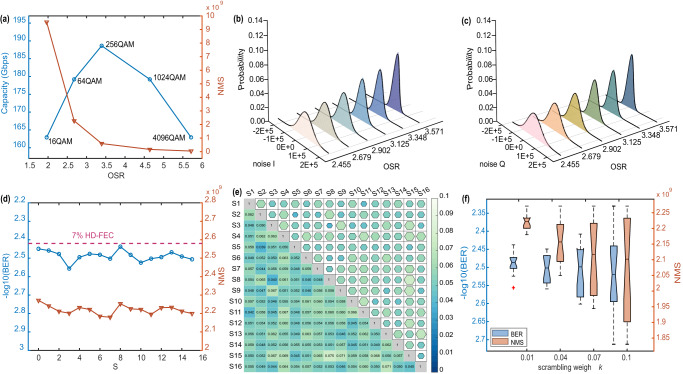
Results of dynamic control and chaotic random number scrambling. **a** The plaintext data capacity and number of masked signals (NMS) versus the oversampling ratio (OSR) when the plaintext bit error ratio (BER) reaches 7% hard-decision forward error correction (HD-FEC). **b**, **c** Probability distribution histograms of the I and Q components masking noise with different OSRs. **d** The BER and NMS of 64-quadrature amplitude modulation (QAM) signal with different scrambling combinations. **e** The Pearson’s correlation coefficient of masking noise between different scrambling combinations. **f** The boxplot of BER and NMS under different scrambling weigh *k*.

As mentioned above, the SNR of the plaintext signal is positively correlated with the OSR. Figure 7a shows the plaintext capacity and NMS versus OSR when the plaintext BER reaches 7% HD-FEC (raw data see Supplementary Data 3). We assume that the system can transmit error-free 40 G-Baud DP-16QAM signals without other overheads. The capacity of plaintext signal is (40 G-Baud × log2(M) bit/symbol × 2 pol)/OSR/(1 + 7% FEC for M-QAM), where the OSR is the minimum value required when the BER of M-QAM signal reaches 7% HD-FEC. Table 2 shows the parameter setups and the results of digitization for M-QAM signals with different OSRs. Although the high-order signal transmission supported by high SNR will increase the spectrum utilization efficiency, the raising OSR will compress the signal bandwidth. It shows that a maximum capacity in efficiency is obtained for OSR = 3.393 by applying the 256QAM signal. The increase in SNR will decrease the noise level and result in a dropping NMS. To visualize the noise intensity control, the probability distribution histograms of the I and Q components masking noise with different OSRs are shown in Fig. 7b, c, respectively. The standard fitting curves of the histogram are provided for comparison with Gaussian white noise. As for a fixed OSR, the histograms are all generally consistent with the Gaussian fitting curve. Hence, the masking noise can be considered as white noise with a normal distribution. Taking OSR as a variable, the histogram would become thinner and taller as the OSR increases. The width of the normal distribution is defined by the standard deviation. Hence, the NMS decreases when the OSR increases. The conclusion is consistent with the experimental results as shown in Fig. 5a. Therefore, we can change the OSR to realize the regulation of masking strength and communication capacity in different application scenarios.

**Table 2:** Plaintext capacity of different M-QAM signals.

Cases	OSR	M-QAM	Capacity (Gbps)
I	1.964	16	152.3
II	2.679	64	167.4
III	3.393	256	176.3
IV	4.464	1024	167.5
V	5.893	4096	152.2

### Scrambling of chaotic random number sequences improves the randomness of masking noise without affecting system transmission performance

The randomness of masking noise is important for the security of system. We use the chaotic random number sequence scrambling the parameters of the DSM to improve the randomness of the masking noise. Without loss of generality, the OSR is set to 2.679 and the feed-forward coefficients *a*_*1*_, *a*_*2*_, *a*_*3*_, and *a*_*4*_ are scrambled. The scrambling weight *k* is 0.01. The scrambling combinations *S* of four random number sequences are 16 and can be written as:

13 $$S={({b}_{0},{b}_{1},{b}_{2},{b}_{3})}_{b2d},\{S\in N|0 < S < {2}^{4}-1\}$$

The BER and NMS of 64QAM signal with different scrambling combinations are shown in Fig. 7d. The BER and NMS are separately lightly fluctuating around 3.5 × 10^−3^ and 2.2 × 10^9^ under the disturbance of different scrambling combinations. Hence, the effect of the scrambling on the performance of the 64QAM signal is slight. To assess the randomness of masking noise, we study the Pearson’s correlation coefficients of masking noise between different scrambling combinations and show them in Fig. 7e^33,34^. The correlation coefficients are all below 0.1, which indicates that the relationship between the masking noise of different scrambling combinations is weak. Therefore, the scrambling of chaotic random number sequence can enhance the randomness of masking noise and not affect the transmission performance.

We further investigated the effect of random number sequence scrambling weight and frequency on BER and NMS. In Fig. 7f, we draw the boxplot of BER and NMS. The lines of the box from top to bottom are the median of the upper half of the dataset, the median of the dataset, and the median of the lower half of the dataset. The boundary of the lower whisker is the minimum value of the dataset, and the boundary of the upper whisker is the maximum value of the dataset^35^. As the scrambling weight increases, the whiskers of BER and NMS become longer, and the fluctuating ranges of BER and NMS become larger. In other words, a larger scrambling weight would damage the stability of the transmission. Thus, a proper scrambling weight need to be chosen according to the practical situation.

## Conclusion

In this paper, we studied a device-compatible QAM-QNSC scheme based on DSM and optical chaos. The plaintext data was encrypted to extremely high-order modulation ciphertext and masked by IBN. The eavesdropper cannot obtain the correct ciphertext signal for deciphering. Thanks to the DSM, the extremely high-order modulation ciphertext can be delivered through a low-order digital signal without modifying the configuration of the transmitter. The DSM applies two-bit quantization for high-precision conversion by oversampling and noise shaping. The price paid includes faster operation and added digital circuitry, which is getting cheaper with advances in integrated digital circuits. The IBN is more convenient to control than the channel noise. Dynamic control of security performance and plaintext data rate can be realized by adjusting the OSR. The randomness of masking noise is improved by the scrambling of chaotic random number sequence. We demonstrated the scheme in a coherent optical communication system. The transmission of 147.9 Gbps 64QAM-QNSC secure transmission supported by the single carrier 40 G-baud DP-16QAM over 75 km SSMF was achieved. Compared with the traditional QNSC schemes, the encrypted penalty is nearly zero since there are no other external nonlinear factors affecting the transmission ciphertext signal. This scheme has the potential to be used in almost all secure communication architectures such as wireless, optical fiber, and laser communication.

### Supplementary information

Description of Additional Supplementary Files

Supplementary Data 1

Supplementary Data 2

Supplementary Data 3

## Data Availability

The authors declare that the data supporting the findings of this study are available within the paper and its supplementary information files. The source data for Fig. 5, Fig. 6, and Fig. 7 are provided as Supplementary Data 1, Supplementary Data 2, and Supplementary Data 3, respectively.

## Code availability

The code for QNSC-DSM transmission and reception in this study is available in (https://github.com/luohanwen1/NCE-submit-code).
