# Supplementary material for: Device-compatible ultra-high-order quantum noise stream cipher based on delta-sigma modulator and optical chaos
Source: Commun Eng. 2024 Feb 7;3:27. doi: 10.1038/s44172-024-00171-x (PMC10955838; doi:10.1038/s44172-024-00171-x)
Supplement: Supplementary file 1 — Description of Additional Supplementary Files [file 44172_2024_171_MOESM1_ESM.pdf]

Description of Additional Supplementary Files

File name: Supplementary Data 1

Description: The source data for Fig. 5

File name: Supplementary Data 2

Description: The source data for Fig. 6

File name: Supplementary Data 3

Description: The source data for Fig. 7
